# Effects of Explicit Knowledge and Attentional-Perceptual Processing on the Ability to Recognize Fear and Surprise

**DOI:** 10.3390/bs15020166

**Published:** 2025-02-02

**Authors:** Mylène Michaud, Annie Roy-Charland, Mélanie Perron

**Affiliations:** 1 École de Psychologie, Université de Moncton, Moncton, NB E1A 3E9, Canada; annie.roy-charland@umoncton.ca; 2 School of Psychology, Laurentian University, Sudbury, ON P3E 2C6, Canada; mperron@laurentian.ca

**Keywords:** emotional facial expressions, fear–surprise confusion, attentional-perceptual limitation hypothesis, explicit knowledge limitation hypothesis, eye movements

## Abstract

When participants are asked to identify expressed emotions from pictures, fear is often confused with surprise. The present study explored this confusion by utilizing one prototype of surprise and three prototypes of fear varying as a function of distinctive cues in the fear prototype (cue in the eyebrows, in the mouth or both zones). Participants were presented with equal numbers of pictures expressing surprise and fear. Eye movements were monitored when they were deciding if the picture was fear or surprise. Following each trial, explicit knowledge was assessed by asking the importance (yes vs. no) of five regions (mouth, nose, eyebrows, eyes, cheeks) in recognizing the expression. Results revealed that fear with both distinctive cues was recognized more accurately, followed by the prototype of surprise and fear with a distinctive cue in the mouth at a similar level. Finally, fear with a distinctive cue in the eyebrows was the least accurately recognized. Explicit knowledge discriminability results revealed that participants were aware of the relevant areas for each prototype but not equally so for all prototypes. Specifically, participants judged the eyebrow area as more important when the distinctive cue was in the eyebrows (fear–eyebrow) than when the cue was in the mouth (fear–mouth) or when both cues were present (fear–both). Results are discussed considering the attentional-perceptual and explicit knowledge limitation hypothesis.

## 1. Introduction

Research suggests that facial expressions of basic emotions (happiness, sadness, fear, surprise, disgust, and anger) are universally and efficiently recognized (e.g., Ekman, 1992, 2003; Ekman & Friesen, 1986; Ekman et al., 1969; Gosselin & Kirouac, 1995; M. L. Smith et al., 2005). Even at birth, individuals are able to distinguish between an array of facial expressions (Gosselin, 2005). Moreover, the recognition of these aforementioned basic emotional facial expressions is quick and can be accomplished with high accuracy, even under cognitive load (e.g., Tracy & Robins, 2008). According to research with children and adults, happiness is the most easily recognized facial expression (De Sonneville et al., 2002; Gosselin & Larocque, 2000). Using different study techniques, research has revealed that muscular activity in specific regions of the face appears to play a critical role in the recognition of emotional facial expressions (M. L. Smith et al., 2005, 2018; Wegrzyn et al., 2017). For example, the mouth for happiness and the eye and/or eyebrow area for anger, surprise, and fear (Beaudry et al., 2014; Calder et al., 2000). However, among the basic emotions, participants exhibit more difficulty recognizing fear (e.g., Calvo & Lundqvist, 2008; De Sonneville et al., 2002; Matsumoto & Ekman, 1989; Tracy & Robins, 2008; Wiggers, 1982) often confusing it with surprise (Chamberland et al., 2016; Gallant & Roy-Charland, 2021; Gosselin & Kirouac, 1995; Roy-Charland et al., 2014, 2015). The reason behind this difficulty, as well as the associated confusion, remains unclear. While the aforementioned studies and others (e.g., Blais et al., 2012; Jack et al., 2014; F. W. Smith & Schyns, 2009) have proposed explanations to elucidate this confusion, none have examined adults’ explicit knowledge and attentional-perceptual processing in recognizing fear and surprise while systematically manipulating the distinctive cues between them.

One explanation for the confusion between fear and surprise is attributed to attentional-perceptual limitations in discriminating facial patterns because of visual similarities between these two emotions (e.g., Camras, 1980; Roy-Charland et al., 2014, 2015; Wiggers, 1982). This hypothesis suggests that the confusion between fear and surprise could be attributed to the difficulty in perceiving distinctive features between them or a lack of attention on these critical features that would favor recognition (Roy-Charland et al., 2014, 2015). In effect, these emotions share numerous muscle activations (action units (AUs)), such as raising of the eyebrows (AUs1+2 in the Facial Action Coding System (FACS), Ekman et al., 2002), raising of the upper eyelids (AU5) and jaw drop (AU26). However, two muscle activations that are seen in fear are also seen in surprise: the brow lowerer (AU4) and lip stretcher (AU20) (Ekman & Friesen, 1978).

Roy-Charland et al. (2014) tested the attentional-perceptual limitation hypothesis in the recognition of fear and surprise using eye-movement recordings and by manipulating the distinctiveness between expressions. They used three prototypes of fear and one of surprise. The surprise prototype involved the shared muscle activations (AU1+2+5+26). The fear prototypes included the same activations, in addition to the brow lowerer (AU4) for one prototype, the lip stretcher for another (AU20) and both activations for the last. The pictures were presented in pairs. In each pairing, the expression of surprise was presented with one of the three prototypes of fear. Participants answered which of the two expressions depicted fear for half of the trials and surprise for the other half. Results revealed better recognition of fear when it contained both distinctive cues rather than a single distinctive feature in the brow area. However, when the cue was in the mouth only, accuracy was as high as when both distinctive cues were activated. Finally, their results indicated a longer viewing time and more comparisons between expressions when the distinctive cue was in the brow only. Furthermore, when the single distinctive cue was in the brow, participants did not spend more time in the brow area compared to the mouth area. Typically, if a cue is considered more important in the recognition process, more time is spent in that specific area (Beaudry et al., 2014). These results suggest difficulty associated with the processing of the brow lowerer as a single distinctive feature. The authors concluded that their results supported the attentional-perceptual hypothesis when the distinctive cue was in the brow area only.

Another study conducted by Atkinson and Smithson (2020) examined if emotional facial expression recognition varies depending on the feature/region fixated. They found that a fixation in the mouth region improved the classification of fear but not a fixation in the central region; a result that seems to be linked to more effective processing, which is the processing of distinctive cues located in the mouth region as observed by Roy-Charland et al. (2014). Eye-movement data tend to support the idea that the visual processing of the central eyebrow region (which may contain the distinctive fear cue AU4) is not necessarily “privileged” in the recognition of fear. However, the study did not check for the presence of AU4 in the stimuli. Thus, it is possible that the upper region of the fear expressions was similar or identical to that of surprise. Further examination of the attentional-perceptual limitations hypothesis is necessary.

Another explanation is associated with the awareness and knowledge of the distinctive features of fear, which brings us to the explicit knowledge limitation hypothesis. Participants might be aware that the stretching of the mouth (AU20) is a feature of fear but not the frowning of the brow (AU4). Thus, even if they process the region or pay attention to this cue, they might not associate it to the emotion of fear. In effect, while the knowledge of emotion labels improves with age and adults possess the labels (Gosselin, 2005), they might not explicitly know what facial muscle movements compose specific facial expressions. This hypothesis has been proposed and explored in other areas of emotional facial expression processing in adults (e.g., Bartlett et al., 1999; Gosselin et al., 2002; Perron & Roy-Charland, 2013). Studies have shown that adults have difficulty categorizing some action units with regard to specific emotions. For instance, even adults have difficulty categorizing the cheek raiser (AU6), an action unit typically found in authentic expressions of happiness (Bartlett et al., 1999; Gosselin et al., 2002). Others have also found the asymmetry of activation of AUs, which is a sign of the non-authenticity of smiles, as a cue for the latter, even if eye tracking showed differential processing of the two sides of the face (Perron & Roy-Charland, 2013). It was proposed that the lack of knowledge of activation might be an explanation why FACS training (in which you learn how to code action units) is such long and complicated training (e.g., Gosselin et al., 2002). No study has explored this hypothesis with regard to specific expressions of fear and surprise in adults.

Nevertheless, Gosselin and Simard (1999) explored this question in children. They, exactly like Roy-Charland et al. (2014), presented pairs of expressions of fear and surprise but to children (ages 5–6 and 9–10). In addition, they asked participants after each trial why they chose a particular face. They asked if it was because of something special in the brows, the eyes, or the mouth. Overall, the accuracy was higher for the condition involving two distinctive AUs compared to the condition involving only one. Furthermore, younger children could not identify unique AUs for the emotions. Finally, the authors identified a bias for the mouth in both age groups, which they explained by either being methodology-related (the order in which regions were presented to children) or the fact that the lip stretcher is an AU unique to fear, while the brow lowerer is also present in expressions of sadness and anger. Since both children and adults have been found to confuse fear and surprise, it is expected that, if the explicit knowledge limitation hypothesis proves to be true, adults are also unaware of the distinctive cues (Gosselin & Simard, 1999).

The current study examined both the attentional-perceptual limitation and explicit knowledge limitation hypotheses in the confusion between fear and surprise. According to these hypotheses, the confusion would be attributed to the lack of knowledge (explicit knowledge) or the difficulty in directing attention and perceiving (attentional-perceptual) the critical distinctive cues essential in recognizing fear and surprise while controlling for the presence of the distinctive cues (AU4, AU20 or AU4+20).

## 2. Materials and Methods

***Participants*:** Fifty-nine individuals (*M* age = 21.86, *SD* = 5.98; 47 women) participated in this study. Power analyses were computed with G Power 3.1.9.7. For repeated measure ANOVAs and the default number of 4 measurements, with an effect size of 0.25, a sample of 24 is sufficient to obtain a power of 0.80. All participants reported normal vision or corrected-to-normal vision.

**Figure 1:**
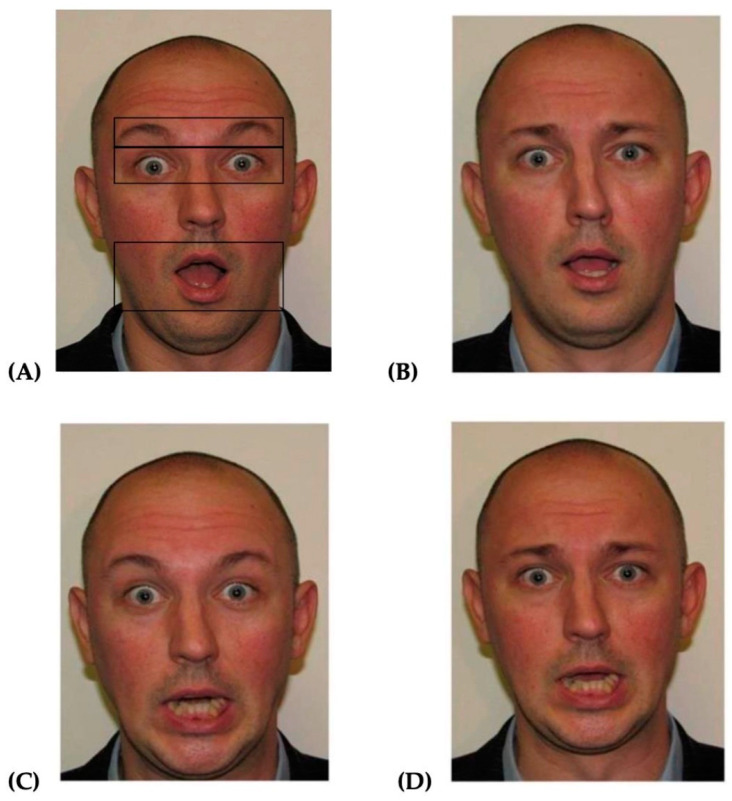
Example of one of the encoders producing the prototype of surprise (**A**) and all three prototypes of fear (**B**–**D**): (**A**) = the prototype of surprise comprised the inner brow raiser (AU1), the outer brow raiser (AU2), the upper lip raiser (AU5), and the jaw drop (AU26); (**B**) = the prototype of fear that includes the action units of surprise (**A**) and the brow lowerer (AU4); (**C**) = the prototype of fear that includes the action units of surprise (**A**) and the lip stretcher (AU20); (**D**) = the prototype of fear that includes the action unit of the prototype of surprise and of both the brow lowerer (AU4) and the lip stretcher (AU20). The zones used to compute the proportion of time in the eyebrows, eyes and mouth are superimposed on (**A**) as an example.

***Materials*:** The three prototypes of fear and one of surprise were taken from Roy-Charland et al. (2014) and were produced by 2 male and 4 female Caucasian encoders. The prototypes of surprise and fear shared the same action units (AU1+2+5+26), except the latter included the brow lowerer (AU4), the lip stretcher (AU20) or both (see Figure 1). The faces were approximately 8.53° degrees by 11.31° in visual angles.

***Eye-Movement Apparatus*:** The SR Research EyeLink II system was used to record participants’ eye movements during the experimentation. Default settings were used (see Perron & Roy-Charland, 2013 for identical settings).

***Procedure*:** Participants were tested individually in a 45 min session. They were seated at approximately 60 cm from the screen. The experimentation comprised 36 trials, 18 of fear (6 per prototype) and 18 of surprise (6 pictures of the facial expression of surprise were shown 3 times), which were presented one at the time in a randomized order. Each trial began with a fixation dot. The image presentation was only initiated when participants fixated the dot. Viewing duration and trial initiation were controlled by the participants. When ready to answer verbally, whether the expression was fear or surprise, participants pressed the left mouse button, which made the picture disappear. To make the next picture appear, participants fixated the dot. To ensure attention was not triggered to any region, eye-movement data were collected prior to the explicit knowledge questions. Immediately after their response, the same picture reappeared and participants had to say *yes* or *no* to the following question: “Do you think that it is the (mouth, nose, eyebrows, eyes, or cheeks) that allowed you to say that this person is expressing fear (or surprise)?”. Participants had to answer that same question for each region and were able to answer ‘’yes’’ to more than one region. The region order was counterbalanced using a Latin square. While we understand that this is not a perfect measure, it was used to be able to compare with Gosselin and Simard (1999), which is the only other study to our knowledge to explore the explicit knowledge question in the confusion between fear and surprise.

***Data analysis*:** Eye movements were scored using the SR Research EyeLink Data Viewer. The proportion of time spent in each area (the eyes, the mouth, and the eyebrows) was computed by dividing the time spent in the specified area by the total time spent on the stimulus. At least one fixation had to occur in an area for the program to record an observation, resulting in an empty cell if that was the case. The eye and eyebrow zones were approximately 4.76 degrees by 0.95 degrees in visual angles, and the mouth was 4.76 degrees by 2.86 degrees, as they were adjusted for each stimulus. As previously mentioned, the viewing time was controlled by the participant, rending proportions more appropriate than the total dwell time since it controls for the variations in viewing times. Nevertheless, analyses were also computed on total dwell time. The total viewing time for each trial was computed by adding all fixation durations from the onset of the stimulus presentation to its disappearance (as controlled by the viewer). No limit was set in the upper or lower bounds of total viewing time. Normality was examined for accuracy and viewing time measures as a function of conditions. Skewness was within the acceptable range for all measures and conditions, while kurtosis was within the acceptable range for all but three conditions for which they were moderately high. Nevertheless, studies have shown that results from ANOVAs are not significantly influenced by small deviations of normality (Blanca et al., 2023; Schmider et al., 2010). To assess if the participants were able to perceive and discriminate the relevant from the irrelevant areas to identify the emotions, we computed a discriminability measure for accurate trials. The relevant areas were those with essential action units to identify the prototypes of fear and surprise (eyebrow, eye, mouth), and the irrelevant were the areas without them (nose, cheeks). The measure was simply the proportion of time spent in the relevant minus the irrelevant areas. A score of −1 indicated that participants spent all the time on irrelevant areas, a score of 0 indicated equal time spent on irrelevant and relevant areas and a score of 1 indicated that participants spent all the time on the relevant areas. Finally, to investigate explicit knowledge for each prototype, we explored the yes or no answers to the questions whether a specific zone (eyebrows, eyes, nose, cheeks, and mouth) had helped the participants identify the emotion presented. We computed a discriminability measure with the proportion of a yes answer for relevant interest areas (eyebrows, eyes, mouth) minus the proportion of a yes answer for irrelevant interest areas (nose, cheeks) for accurate trials. In this case, a score of -1 indicated an absence of knowledge, while a 1 indicated a perfect knowledge of relevant interest areas. Data are available on the OSF website (see Data Availability Statement). For all analyses, an alpha level of 0.05 was selected.

## 3. Results

### 3.1. Accuracy

Means and standard deviations for correct responses as a function of prototype are presented in Table 1 (please see Figure 1 for visual reference; A = surprise [AU1+2+5+26], B = fear–eyebrow [AU1+2+5+26 + AU4], C = fear–mouth [AU1+2+5+26 + AU20]), D = fear–both [AU1+2+5+26 + AU4 + AU20]). The main effect of prototypes was significant, F (2.197, 127.416) = 74.11, *p* < 0.001, $\eta_{p}^{2}$ = 0.56. Post hoc comparison revealed that the fear–both prototype was the most accurately identified (all *p*s < 0.001, all ds > 0.89), followed by surprise and fear–mouth (between fear–mouth and surprise, *p* = 0.36), and then fear–eyebrow (all *p*s < 0.001, all ds > 1.62).

Analyses were also computed for each prototype to compare accuracy with the chance level (0.50). For surprise, fear–both and fear–mouth, accuracy was better than chance, respectively, t(58) = 17.47, *p* < 0.001, t(58) = 25.47, *p* < 0.001, t(58) = 8.95, *p* < 0.001 but not for fear–eyebrow, t(58) = −1.15, *p* = 0.25. Signal detection analyses were utilized with fear as the signal. Results revealed strong signals for fear–both (d’ = 1.78) and fear–mouth (d’ = 1.24) but not for fear–eyebrow (d’ = 0.41).

### 3.2. Total Viewing Time

The effect of prototype was significant, F (3, 174) = 8.52, *p* < 0.001, $\eta_{p}^{2}$ = 0.13, for total viewing time (see Table 1). Post hoc comparison revealed that participants spent less time viewing fear–both than surprise and fear–eyebrow (*p*s < 0.001, ds > 0.20) and less time viewing fear–mouth than fear–eyebrow (*p* = 0.013, d = 0.21). None of the other comparisons were significant (all *p*s > 0.16).

#### Eyebrows, Eyes, Mouth

The proportion of time spent on eyebrows, eyes, and mouth as a function of prototype was assessed in separate analyses (Table 2). For all three zones, respectively, eyebrows, eyes and mouth, there was an effect of the prototype, F (3, 174) = 5.12, *p* < 0.003, $\eta_{p}^{2}\;$ = 0.08, F (3, 174) = 7.64, *p* < 0.001, $\eta_{p}^{2}$ = 0.12, F (3, 174) = 35.51, *p* < 0.001, $\eta_{p}^{2}$ = 0.38. Post hoc comparison revealed more time in the eyebrows for fear–eyebrow and surprise, respectively, than for fear–both (*p* = 0.028, d = 0.32; *p* = 0.049, d = 0.25) and fear–mouth (*p* = 0.002, d = 0.38; *p* < 0.001, d = 0.31). No difference was observed between fear–eyebrow and surprise (*p* = 0.27) or fear–both and fear–mouth (*p* = 0.55). The same pattern was observed for the eye zone: more time for fear–eyebrow and surprise than for fear–both (*p* < 0.001, d = 0.33; *p* = 0.001, d = 0.24) and fear–mouth (*p* = 0.003, d = 0.33; *p* = 0.004, d = 0.24), and no difference between fear–eyebrow and surprise (*p* = 0.36) or fear–both and fear–mouth (*p* = 0.75). Oppositely, results revealed more time (all *p*s < 0.001, d = 0.42) in the mouth for fear–both and fear–mouth (no difference between the two; *p* = 0.59) than for surprise and fear–eyebrow (no difference between the two; *p* = 0.15).

### 3.3. Explicit Knowledge

A discriminability measure was computed with the proportion of a yes answer for relevant interest areas (eyebrow, eye, mouth) minus the proportion of a yes answer for irrelevant interest areas (nose, cheeks) for accurate trials. Results revealed a significant effect of the prototype, F (3, 174) = 11.66, *p* < 0.001, $\eta_{p}^{2}$ = 0.17. Participants were better at discriminating relevant areas for surprise than fear–both and fear–eyebrows (all *p*s < 0.001, d = 0.46) and for fear–mouth than fear–both and fear–eyebrows (all *p*s < 0.005, d = 0.33). No other significant differences were observed (*p*s > 0.14). All discriminability scores were superior to 0 (confirmed by one sample *t*-tests; all *p*s < 0.001). The higher the score, the higher the explicit knowledge for cues in this area for the recognition of the emotion.

#### Eyebrows, Eyes, Mouth

The proportion of yes answers for eyebrows, eyes, and mouth as a function of each prototype was assessed separately for each zone (Table 2). The higher the score, the higher the explicit knowledge for cues in this area for the recognition of the emotion. For eyes, the effect of prototypes did not reach significance, F (3, 171) = 2.60, *p* = 0.05, $\eta_{p}^{2}$ = 0.04, meaning the eyes were deemed as equally important for each prototype. A significant effect was found for eyebrows, F (3, 171) = 5.81, *p* = 0.001, $\eta_{p}^{2}$ = 0.09, and mouth, F (3, 171) = 7.55, *p* < 0.001, $\eta_{p}^{2}$ = 0.12. Post hoc comparison for eyebrows revealed that participants identified the latter as more important for fear–eyebrow than for fear–both (*p* = 0.032, d = 0.29) and fear–mouth (*p* = 0.022, d = 0.41) and more for surprise than for fear–both (*p* = 0.003, d = 0.38) and fear–mouth (*p* < 0.001, d = 0.50). No difference was observed between fear–eyebrow and surprise (*p* = 0.66) or fear–mouth and fear–both (*p* = 0.20). For mouth, post hoc comparison revealed that participants reported that the mouth was more important for fear–both than for fear–eyebrow (*p* = 0.003, d = 0.76) and surprise (*p* = 0.006, d = 0.62) and more for fear–mouth than for fear–eyebrow (*p* = 0.004, d = 0.61) and surprise (*p* = 0.002, d = 0.40). No difference was observed between fear–both and fear–mouth (*p* = 0.66) or surprise and fear–eyebrow (*p* = 0.12).

### 3.4. Correlations

A series of correlations was computed between accuracy and, respectively, total viewing times, the proportion of time spent in the eyebrows, eyes and mouth for each type of prototypes. For fear–both, the more time participants spent looking at the mouth, the more accurate they were (r = 0.29, *p* < 0.028). For fear–eyebrows, the longer they spent viewing the stimuli, the more accurate they were (r = 0.26, *p* < 0.047). None of the other correlations were significant (all rs < 0.17, all *p*s > 0.20).

A second series of correlations was computed between the proportion of time spent in a zone and the proportion of yes answers they gave with regard to the importance of this zone in their explicit knowledge. None of the correlations were significant (all rs < 0.23, all *p*s > 0.09).

## 4. Discussion

The present study explored the confusion between fear and surprise considering the explicit knowledge limitation and attentional-perceptual limitation hypotheses that are presented in the literature as distinctive potential explanations for difficulty in recognizing the expressions. According to these hypotheses, the confusion could be attributed either to a lack of knowledge (explicit knowledge limitation hypothesis) or to difficulty in directing attention and/or perceiving (attentional-perceptual limitation hypothesis) the critical distinctive cues between fear and surprise, which are essential to their recognition. However, participants would require perceiving and know the critical distinctive cues, and they should spend more time and judge the areas with the critical cues as more important than the areas without them to ultimately produce an accurate response.

Fear comprising both distinctive cues was the most quickly and accurately recognized from the other prototypes. Accuracy between prototypes can be summarized as follows: fear–both > surprise = fear–mouth > fear–eyebrow. Like Gosselin and Simard (1999), we found that the presence of two distinctive cues offered better accuracy than just one, but this time in adults. However, our accuracy results differed from those of Roy-Charland et al. (2014) as they found no sign of additive value to the combination of both distinctive cues in comparison to a single cue in the mouth. This disparity could possibly be attributed to the difference in methodology or the fact that the latter study had a ceiling effect. In their study, participants had to select between two pictures presented in pairs (which arguably emphasizes discrimination), whereas, in our study, only one picture at a time was presented, making the task harder and more focused on recognition (Gosselin, 2005). Also, Roy-Charland et al. (2014) obtained a ceiling effect (superior to 0.96), thus reducing the chance to detect a difference between high performance on both cues and the cue in the mouth only.

***Explicit knowledge limitation hypothesis*:** According to this hypothesis, confusion between fear and surprise could occur because of a lack of knowledge regarding distinctive cues between the two emotions. Overall, our research provided support against the explicit knowledge limitation hypothesis as it suggested that participants showed knowledge of important cues. They judged the eyebrows as being more important for fear–eyebrow than for fear–mouth, but also than for fear–both. Unlike Gosselin and Simard’s (1999) research where children were found to overestimate the importance of the mouth area but not the differences in the brow and eye regions, our results suggested that the eyebrows were judged more important for fear–eyebrows than the other fear prototypes. Nevertheless, our results still indicated a preferential role of the mouth since, for fear–both, participants judged this zone equally important as for fear–mouth, but the eyebrow area is judged as less important for fear–both compared to fear–eyebrow.

***Attentional-perceptual limitation hypothesis*:** According to this hypothesis, confusion between the two emotions could be explained by the difficulty in directing attention and perceiving the critical distinctive cues essential in their differentiation. Eye-movement results partly provided support against the attentional-perceptual limitation hypothesis. The viewing pattern for fear prototypes suggested that participants directed their attention to the relevant cues. For instance, participants spent more time in the eyebrows for fear–eyebrow than fear–mouth and more time in the mouth for fear–mouth than fear–eyebrows, suggesting that participants direct their attention to the cues available to them. However, even when participants have all the time they want and even when they direct their attention to relevant areas, their performance for fear with a single distinctive cue in the eyebrow was poor. In other words, it is not the time spent in the critical region that seems to explain poor performance in the recognition of fear–eyebrow. While the “attentional” portion of the hypothesis might not be supported, the current results cannot exclude its “perception” portion. In other words, it is possible that participants paid attention to the distinctive cues but were not able to perceive the subtle cues. For example, participants may have difficulty in distinguishing AU4 (brow lowerer) when the prototypes also contain AU1 (inner brow raiser) and AU2 (outer brow raiser). Future research should propose methodologies that allow for the clear distinction between attention and perception.

The fact that eye-movement patterns did not explain the different degree of precision between surprise and fear–eyebrow could perhaps be partly explained by the high intensity of facial stimuli (Vaidya et al., 2014). Research has shown that emotional facial expression recognition could not be predicted based on eye movements for expressions of extreme intensity. Instead, results showed that the fixation pattern rather predicts the recognition of subtle emotional expressions (Vaidya et al., 2014). In the presence of intense expressions, data seem to suggest that the weight given to certain regions in terms of fixation does not seem to play a major role in recognition. However, it would be wrong to claim that, when emotional facial expressions are intense, exploration and attention to regions are uniform and similar across expressions and play no role. Roy-Charland et al. (2014) observed that participants tended to have more visual saccades and spent more time in the eye/brow region between two simultaneously presented expressions (one of surprise and one of fear) when the distinctive cue was in the eyebrows but not when it was in the mouth or when fear contained the two distinctive cues. The analyses also revealed that participants spent more time in the mouth when there was a distinctive cue in this region than when the distinctive cue was in the eyebrow only. Roy-Charland et al. (2015) also observed longer viewing times when the distinctive cue was in the eyebrows only compared to the mouth or both regions. Moreover, 3–5-year-olds, 9–11-year-olds, and adults had more visual saccades between expressions when the distinctive cue for fear was in the eyebrows only. These results were observed with intense expressions. A more appropriate explanation for the differences between the current study and Roy-Charland et al. (2014) might be the fact that the tasks are different. In effect, studies have shown that variations in the specific methodologies can influence the pattern of visual processing of emotional facial expressions (e.g., M. L. Smith & Merlusca, 2014). Nevertheless, future studies should explore these differences systematically.

***Limitations and Future research*:** Our study is not without limitations. For instance, our research utilized static stimuli, which provides no context or movement and is therefore not representative of a real-world situation. Future research could use dynamic stimuli, which could provide the viewer with a timing of muscle activations for each prototype of the facial expression. Furthermore, the requirement of the current task with only two labels for possible answers and two emotions as prototypes artificially inflates accuracy rates compared to recognition tasks with other emotions and more labels. Finally, our sample had an overrepresentation of women (80%) compared to men. Because of gender differences in the visual strategies underlying face perception (Coutrot et al., 2016; Hall et al., 2010), it could be argued that our results are to be considered carefully regarding generalizability.

## Figures and Tables

**Table 1:** Means and standard deviations for accuracy proportions, total viewing times, and explicit knowledge according to prototype.

		Prototypes		
	Surprise	Fear–Both	Fear–Mouth	Fear–Eyebrows
	M (SD)	M (SD)	M (SD)	M (SD)
Accuracy	0.81 (0.14)	0.92 (0.13)	0.77 (0.24)	0.47 (0.13)
Total viewing time (ms)	5112 (2827)	4190 (2475)	4745 (3064)	5361 (2862)
Explicit knowledge	0.76 (0.18)	0.66 (0.23)	0.74 (0.22)	0.67 (0.21)

**Table 2:** Means and standard deviations of viewing time proportions and explicit knowledge proportions according to prototype and interest area.

			Prototypes		
		Surprise	Fear–Both	Fear–Mouth	Fear–Eyebrows
		M (SD)	M (SD)	M (SD)	M (SD)
Viewing time					
	Eyes	0.22 (0.14)	0.19 (0.11)	0.19 (0.11)	0.23 (0.13)
	Eyebrows	0.10 (0.07)	0.08 (0.09)	0.08 (0.06)	0.11 (0.10)
	Mouth	0.20 (0.09)	0.24 (0.10)	0.25 (0.09)	0.19 (0.10)
Explicit knowledge					
	Eyes	0.91 (0.16)	0.93 (0.17)	0.94 (0.14)	0.95 (0.12)
	Eyebrows	0.87 (0.18)	0.79 (0.24)	0.74 (0.34)	0.86 (0.25)
	Mouth	0.96 (0.11)	1.0 (0.02)	0.99 (0.04)	0.92 (0.19)

## Data Availability

Data for the current study are available through open access at https://osf.io/hzgr6/?view_only=9cbe348ff02741dfbe9a263b1b82690f (accessed on 29 January 2025).
